# Survey the Occurrence of *Globocephalus urosubulatus* (Nematoda: Ancylostomatidae) in Wild Boars (*Sus scrofa*) in the State of São Paulo, Brazil

**DOI:** 10.3390/vetsci11080370

**Published:** 2024-08-12

**Authors:** Michel dos Santos Pinto, João Alfredo Biagi Camargo Neto, Maria Julia Hernandes de Freitas, Bárbara Fuzetto Florentino, Natália de Souza Sapatera, Fernando Paiva, Alex Akira Nakamura, Daniela Bernadete Rozza, Simone Baldini Lucheis, Katia Denise Saraiva Bresciani

**Affiliations:** 1 Faculdade de Medicina Veterinária, Universidade Estadual Paulista (UNESP), Araçatuba 16050-680, SP, Brazil; joao.alfredo@unesp.br (J.A.B.C.N.); mjh.freitas@unesp.br (M.J.H.d.F.); katia.bresciani@unesp.br (K.D.S.B.); 2 Instituto de Biociência, Universidade Federal do Mato Grosso do Sul (UFMS), Campo Grande 79070-900, MS, Brazil; fernando.paiva@ufms.br; 3 Faculdade de Medicina Veterinária e Zootecnia, Universidade Estadual Paulista (UNESP), Botucatu 18618-687, SP, Brazil; simone.b.lucheis@unesp.br

**Keywords:** helminths, parasites, swine

## Abstract

**Simple Summary:**

Free-ranging wild boars are considered exotic animals and cause various ecological, economic and health-related damages in Brazil. With the increasing trend of outdoor pig farming, the chances of domestic animals coming into contact with wildlife have risen, leading to a greater risk of disease transmission. Studies on gastrointestinal parasites in wild boars in Brazil are scarce, so we investigated the occurrence of parasitic helminths in these animals. In our study, a total of 10 animals—5 males and 5 females of different ages—were examined. After these animals were culled by control and hunting teams, their digestive systems were collected and examined. A total of 2750 helminths were collected from the small intestine of all animals, and after evaluating their morphologies, we identified them as *Globocephalus urosubulatus*. The morphological characteristics observed in the nematodes correspond to those previously described; however, we found that the copulatory bursa was asymmetrical, a characteristic that has not yet been recorded in the literature.

**Abstract:**

Although previous studies have characterized the helminth fauna of wild boars kept in captivity in Brazil, records on these helminths in free-ranging animals are still scarce. In view of this, we aimed in our work to investigate the occurrence and morphological and morphometric characteristics of gastrointestinal helminths in wild Sus scrofa from the northwest region of the State of São Paulo, Brazil. The digestive systems of 10 animals (5 males and 5 females of different ages) were used in this study. Each anatomical segment was washed and sieved under running water, and the helminths were separated and identified using light and scanning electron microscopy, according to their morphological characteristics. A total of 2750 (1152 males and 1598 females) nematode specimens were collected from the small intestine of these wild boars, and all of them presented the morphological characteristics of *Globocephalus urosubulatus*. However, one characteristic is of particular interest because it has not yet been reported in the literature: a marked asymmetry between the lobes and their respective rays of the copulatory bursa, with the left one being larger than the right one. In this research, we identified the presence of *G. urosubulatus* in all the examined free-ranging wild boars and reported for the first time in the literature the asymmetry in the copulatory bursa.

## 1. Introduction

The free-ranging wild boar (*Sus scrofa*, Linnaeus 1758) is an invasive species in Brazil and in several regions of the world [1]. Of Eurasian origin, this animal was taken to the Americas during the period of great navigation, and due to the lack of a natural predator, it spread throughout the continent [2].

In this scenario, the introduction of wild boars in Brazil is uncertain and fragmented. The first record of entry of these animals, from Uruguay, occurred in Rio Grande do Sul in the last century. The second report was the importation of wild boars from Canada and France for commercial breeding in the 1990s and 2000s. However, the failure of this activity, which at the time showed promise, led to the release of these animals intentionally or not to the environment. As a result, wild boars began to cause great ecological, economic and health damage to the country [3]. 

Domestic and wild pigs are constantly affected by helminthiasis [4]; normally, these infections remain subclinical for a long period, but they can also lead to these animals dying in cases of severe infection [5,6].

Although previous studies have characterized the helminth fauna of wild boars in captivity in Minas Gerais [7], São Paulo [8] and Rio Grande do Sul [9], investigations into free-ranging *Sus scrofa* in Brazil are still scarce, with only one registration in the northeast region of the State of São Paulo [10]. 

Thus, since there is a gap regarding the parasitic fauna in free-ranging wild boars in Brazil, we aimed in our study to identify the occurrence and morphological and morphometric characteristics of gastrointestinal helminths in wild *Sus scrofa* from the northwest region of the State of São Paulo, Brazil.

## 2. Materials and Methods

### 2.1. Study Population 

In the present study, the gastrointestinal tract and attached glands of 10 free-ranging wild boars (5 males and 5 females with varying ages) slaughtered by control and hunting teams in the northwest region of the State of São Paulo, Brazil, were examined. They inhabited farm areas with crops and cattle and horse breeding, in addition to sharing the environment with other wild animals.

### 2.2. Sample Collection and Processing

After slaughter, the entire digestive system was collected, stored in a plastic bag, stored in isothermal boxes with flexible ice packs (Gelox^®^) and sent to the laboratory for investigation. Thus, the stomach, small intestine, large intestine, pancreas and liver of each animal were analyzed. The gastrointestinal contents of each anatomical segment were washed under running water, sieved in sieves with openings of 0.30 mm (mm) and placed in duly identified bottles containing 70 GL ethyl alcohol. Multiple incisions were made and macroscopic examination was performed in the liver and pancreas of each animal to investigate trematodes. All helminths were then collected using a stereoscopic microscope and packaged in bottles containing 70 GL ethyl alcohol. After that, they were gradually transferred to 80 and 90 GL ethyl alcohol for at least 24 h and then fixed and stored in absolute ethyl alcohol.

### 2.3. Helminths Identification

For identification, nematodes were clarified with a 95% alcoholic phenol solution, separated by sex, quantified and identified according to their morphology [10,11].

### 2.4. Morphological and Morphometric Analysis

For morphological and morphometric analysis, 20 specimens (10 males and 10 females) were examined under a light microscope (Olympus) with an Olympus SC 100 camera and an image capture system.

### 2.5. Scanning Electron Microscopy

Scanning electron microscopy was performed according to [12]. A total of 10 specimens (5 males and 5 females) were selected for scanning electron microscopy. These were immersed in hexamethyldisilazane (Cat. Number 440191; Sigma-Aldrich™, St. Louis, MO, USA) for 10 min and deposited on conductive carbon tapes 12 mm in diameter (PELCO TABS™; Ted Pella^®^, Redding, CA, USA), previously adhered to metal stubs. The dimensions were 12.7 × 12.7 mm (Ted Pella^®^, Inc., Redding, CA, USA), and microscopic images were documented by a scanning electron microscope (Hitachi^®^ model TM3000TM; Tokyo, Japan) in analy mode.

### 2.6. Results Analysis

The results of this study were analyzed by determining the ecological descriptors of parasitism according to Margolis [13], and the morphometric data were compared with those published by several authors [10,11,14].

## 3. Results

### 3.1. Epidemiology

In our study, of the ten animals investigated, all (100%) presented helminthic infections. A total of 2750 (1152 males and 1598 females) nematode specimens were collected from the small intestinal contents of these individuals. In the macroscopic analysis performed after multiple cuts were made in the adnexal glands of the animals investigated, no parasitic forms were found. Moreover, we did not detect any examples of helminths in the stomach and large intestine of these wild boars.

All nematode specimens collected and examined in this research showed morphological characteristics compatible with *Globocephalus urosubulatus* (Alessandrini, 1909), and the Ecological descriptors of parasitism are described in Table 1.

The animals evaluated had no lesions in the small intestine, and all of them were in great body condition and appeared to be healthy.

### 3.2. Morphological Characteristics

**Figure 1:**
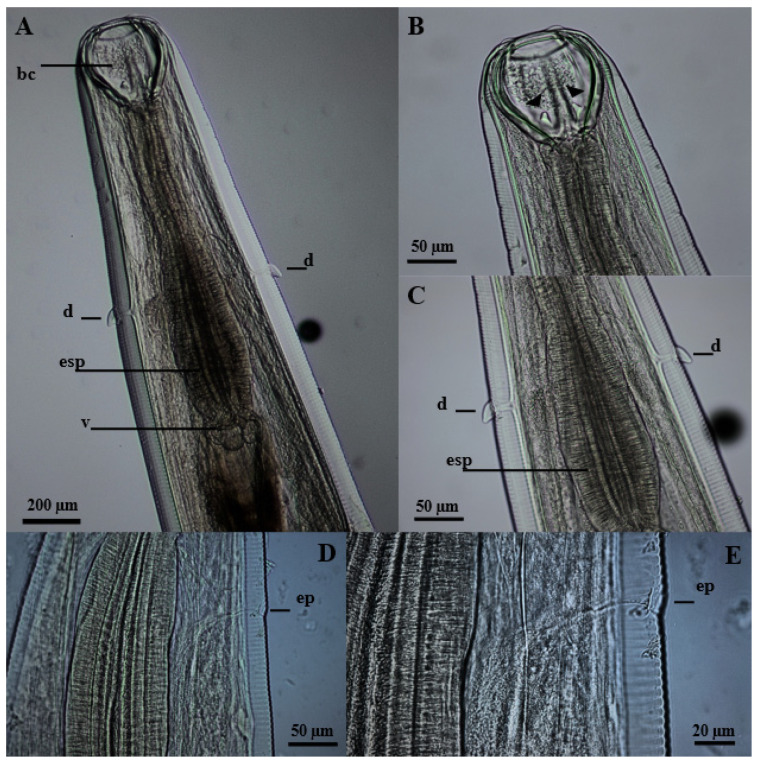
Anterior region of *Globocephalus urosubulatus* (Alessandrini, 1909) recovered from 10 free-ranging wild boars in the northwest region of the State of São Paulo, Brazil. (**A**) Ventro-dorsal view of the agent, highlighting the buccal capsule (**bc**), the lateral deirids (**d**), clavicular esophagus (**esp**) and the esophageal valve (**v**). (**B**) Ventro-dorsal view of the cephalic region showing two subventral teeth (arrows). (**C**) Ventro-dorsal view of the parasite demonstrating the lateral deirids (**d**) and the claviform esophagus (**esp**). (**D,E**) Right latero-lateral view of the nematode showing the excretory pore (**ep**).

**Figure 2:**
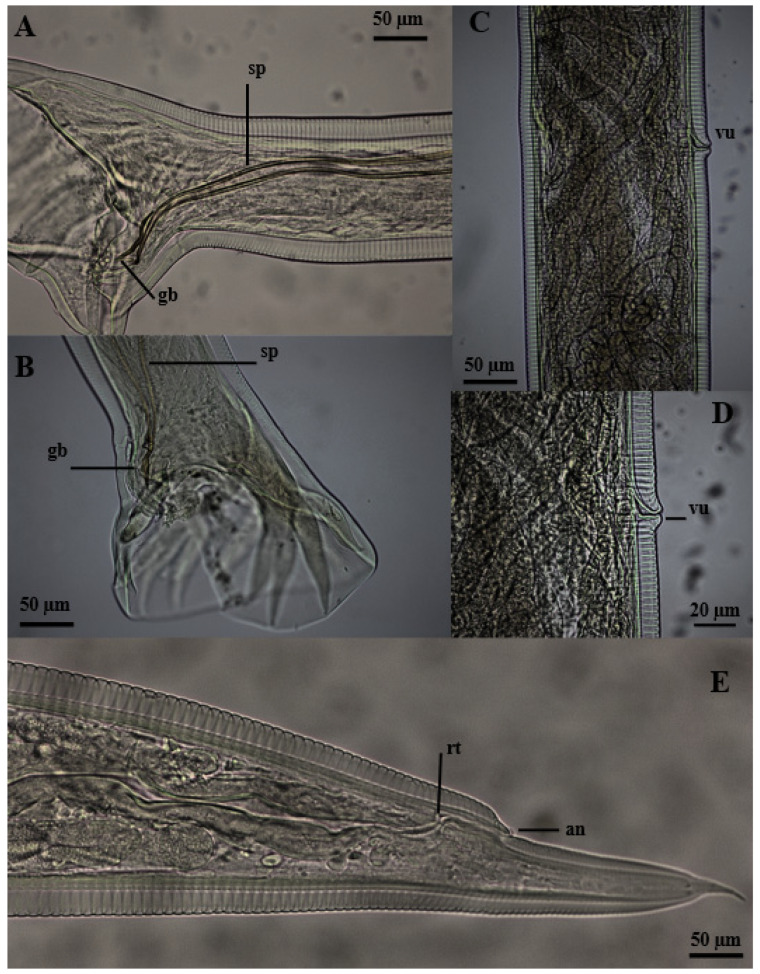
Posterior region of *Globocephalus urosubulatus* (Alessandrini, 1909) recovered from 10 free-ranging wild boars in the northwest region of the State of São Paulo, Brazil. (**A**,**B**) Right and left latero-lateral view, respectively, of a male specimen showing the spicules (**sp**), the gubernaculum (**gb**) and the rays of the copulatory bursa. (**C**,**D**) Right latero-lateral view of a female specimen showing the edges of the vulva (**vu**) in the ventral region. (**E**) Right latero-lateral view of the final portion of the female highlighting the anus (**an**) and rectum (**rt**).

**Figure 3:**
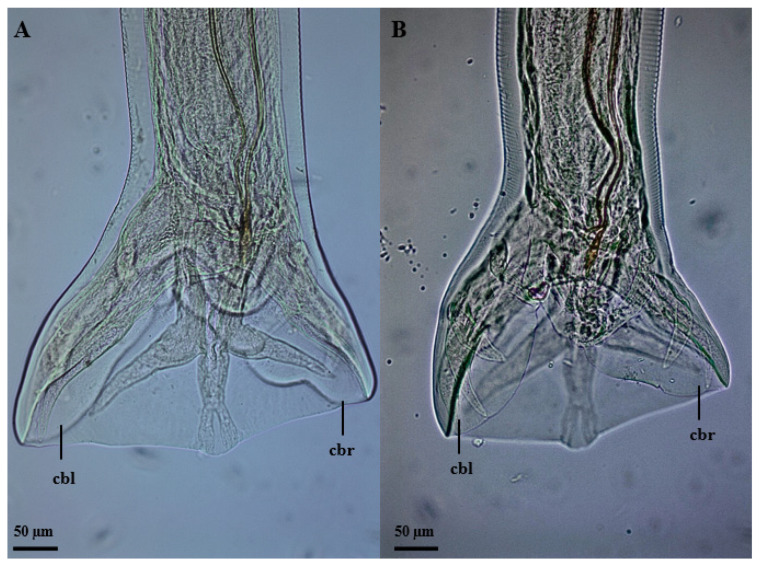
Posterior region of *Globocephalus urosubulatus* (Alessandrini, 1909) recovered from 10 free-ranging wild boars in the northwest region of the State of São Paulo, Brazil. (**A**,**B**) Dorso-ventral view of the posterior region of male specimens, highlighting the asymmetry in the copulatory bursa, with greater development of the left lobe and respective rays (**cbl**) when compared to the right side (**cbr**).

**Figure 4:**
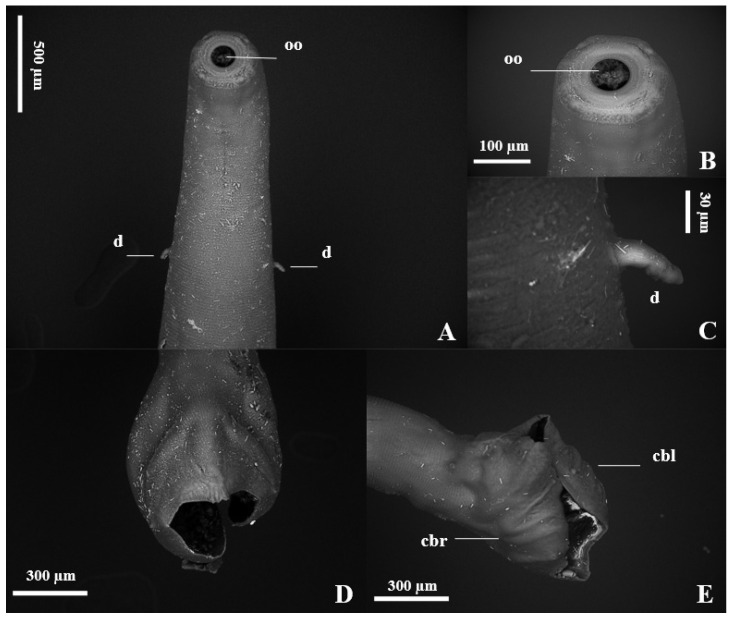
Scanning electron microscopy of *Globocephalus urosubulatus* (Alessandrini, 1909) recovered from 10 free-ranging wild boars in the northwest region of the State of São Paulo, Brazil. (**A**–**C**) Ventral view of the anterior portion of the nematode, highlighting the oral opening (**oo**) and the lateral deirids (**d**). (**D**) Dorsal view of the copulatory bursa highlighting the asymmetry, with the left lobe and its respective rays being larger than the right. (**E**) Right latero-lateral view of the copulatory bursa, highlighting the left (**cbl**) and right lobes (**cbr**).

**Figure 5:**
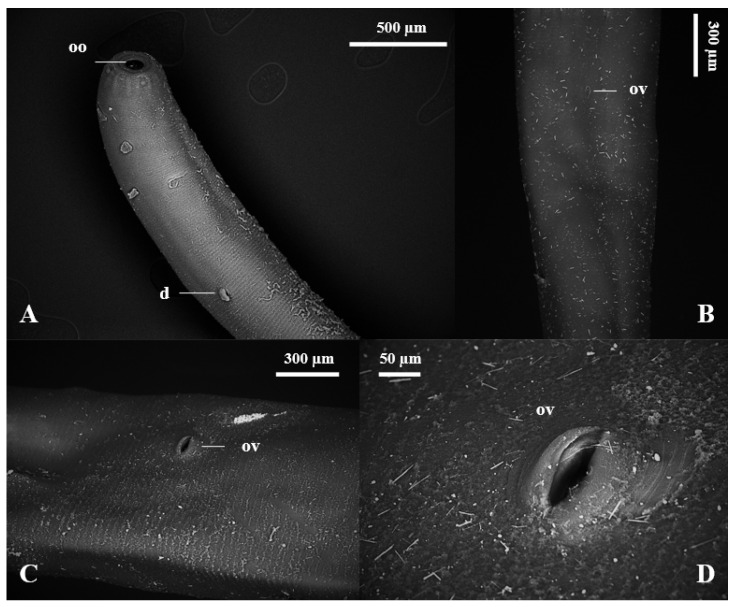
Scanning electron microscopy of the female *Globocephalus urosubulatus* (Alessandrini, 1909) recovered from 10 free-ranging wild boars in the northwest region of the State of São Paulo, Brazil. (**A**) Right latero-ventral panorama of the female, highlighting the oral opening (**oo**) and the right deirid (**d**). (**B**–**D**) Ventral view of the female with the opening of the vulva (**ov**).

The nematodes had a yellowish filiform body, females were larger than males and the morphology of the anterior region was similar between the sexes (Figure 1). The excretory pore was located in the ventral region (Figure 1D,E), and the lateral deirids (cervical papillae) were well developed close to the nerve ring, which surrounded the anterior region of the esophagus (Figure 1A,C). Additionally, it was noted that the cuticle, throughout its entire length, was thick and segmented by thin transverse lines. The oral opening was circular, surrounded by a cuticular ring, and the buccal capsule was well developed, with two subventral teeth close to its base (Figure 1B). The well-developed clavicle-type esophagus had a robust valve (Figure 1A) at the transition to the intestine. Males had similar fusiform-shaped spicules and a thick “half-moon”-shaped gubernaculum at their end (Figure 2A,B). The well-developed copulatory bursa presented evident asymmetry, with the left lobe and its respective rays being more developed when compared to the right, and may even have covered one another (Figure 3 and Figure 4D,E). Females had a conical tail and a more prominent upper lip of the anus (Figure 2E). The vulva was located in the most posterior ventral region, with poorly developed edges (Figure 2C,D and Figure 5C,D).

### 3.3. Morphometric Characteristics of Females

Females measured 6.81 mm (4.73–7.90 mm) and 492.29 µm (423–583 µm) in size and width, respectively. The buccal capsule was 176.81 µm (142–209 µm) long by 146.21 µm (137–163 µm) thick. The muscular esophagus was well developed in the posterior region, with dimensions of 731.21 µm (671–795 µm) × 167.28 µm (141–200 µm). The distances from the anterior end to the nerve ring, deirids and excretory pore were, respectively, 479.51 µm (433–538 µm), 583.86 µm (440–652 µm) and 579.31 µm (409–645 µm). The vulva was located in the ventral region with poorly developed edges and 2.38 mm (1.48–3.0 mm) away from the end of the posterior region. The tail (distance from the anus to the end of the nematode) was 198.45 µm (76–238 µm) long, with mucruns 45.84 µm (25–79 µm) in size.

### 3.4. Morphometric Characteristics of Males

Males had a total length of 5.11 mm (4.70–5.94 mm) and a width of 301.81 µm (286–342 µm). The buccal capsule was developed, with dimensions 149.62 µm (118–190 µm) × 105.94 µm (95–125 µm). The claviform esophagus was 562.27 µm (512–624 µm) in size and had a width of 129.79 µm (112–145 µm). The distances from the nerve ring, deirids and excretory pore to the anterior region were, respectively, 374.06 µm (312–426 µm), 467.19 µm (387–568 µm) and 440.41 (407–531 µm). Similarly, filiform spicules were 516.23 µm (444–573 µm) in size, and the crescent-shaped gubernaculum was 75.72 µm (60–86 µm) long. The copulatory bursa was asymmetrical, with the left lobe and its respective rays being larger (321.25 µm [287.93–393.72 µm]) when compared to the right (220.30 µm [183.13–280.69]). 

The comparison of morphometric characteristics is described in Table 2.

## 4. Discussion

In our study, we were able to identify *G. urosubulatus* in all the free-ranging wild boars investigated, and according to the morphological and morphometric characteristics of the aforementioned agent, under scanning electron microscopy and light microscopy, we found an asymmetry of the copulatory bursa for the first time in the literature. The asymmetrical copulatory bursa has a larger left lobe and respective rays when compared to the right, with an average difference of approximately 100 µm, equivalent to 1/3 of the total size. Several old and current studies on the morphological characteristics and occurrence of *G. urosubulatus* have already been carried out in different locations around the world [10,11,14,15,16,17]; however, this morphological characteristic in the copulatory bursa has not been reported. For a long time, the genus *Globocephalus* underwent different taxonomic proposals, being considered as *Cystocephalus* (1895), *Characostomum* (1902), *Crassisoma* (1909), *Raillietostrongylus* (1923) and *Glococephaloides* (1926). However, the name *Globocephalus* is currently accepted according to the rules of zoological nomenclature [11]. The species *G. urosubulatus* is geographically distributed in Austria, Turkey, New Zealand, France, Germany, Bulgaria, Zaire, Iran, Guyana, Africa, India and America [18]. 

In the free-ranging *Sus scrofa* examined in our research, we observed a 100% occurrence of *G. urosubulatus* and an average abundance and mean intensity of 275. Although the Brazilian Institute of the Environment and Renewable Natural Resources (IBAMA), in 2013, declared wild boars an invasive exotic species in Brazil, as well as harmful animals in terms of One Health, and has allowed the hunting and slaughter of these animals as a form of population control and eradication [19], studies on the helminth fauna of these animals are scarce. The only work on the epidemiology of gastrointestinal nematodes in wild boars in Brazil showed a prevalence of *G. urosubulatus* in 94.3% of the animals investigated, as well as a mean abundance of 215.5 and a mean intensity of 228.6. These data are similar to those found in our research. However, the intensity in our study was greater (17–1931) than that in the other work (1–892) [10].

Our research highlights, for the second time in Brazil, the occurrence of this nematode in free-ranging wild boars, which was identified for the first time in the northeast region of the State of São Paulo [10]. It is worth mentioning that in investigations into the helminth fauna of captive wild boars in this country, this agent was not identified [7,8,9]. These data corroborate epidemiological findings in Poland, characterizing a higher occurrence of *G. urosubulatus* in wild boars when compared to those in captivity [20,21]. 

In our research, the morphometry of the nematodes analyzed demonstrated differences between the sexes. Females and their structures were larger when compared to males. Unlike our findings, in a study of the morphology of *G. urosubulatus* in wild boars in Bulgaria, the helminths were proposed to have the same variations in measurements of the buccal capsule, esophagus and distance between the deirids and nerve ring to the anterior region, regardless of sex [14]. Sizes similar to those found by us were described in helminths collected from the small intestine of a pig in Pará [11], as well as in *G. urosubulatus* in wild boars in Brazil [10], with the exception of the distance from the vulva to the posterior end of the females, which the latter authors describe at a greater distance.

We evidenced a significant occurrence of *G. urosubulatus* in free-ranging wild boars. In recent years, Intensive Free-Range Swine Systems (SISCAL) have grown steadily, due to greater demand for animal products, along with welfare practices. This increases the interaction between production systems and wild boars, increasing the risk of mutual infections [22]. During an investigation on intestinal parasites in *Pecari tajacu* (colored peccary) and *Sus scrofa domesticus* (domestic pig), in the southeast region of Piauí, Brazil, an occurrence of 12.5% (1/8) and 33.33% (9/27) of *G. urosubulatus* was found in these animals, respectively [23]. This demonstrates the sharing of this agent between wild and domestic pigs.

The pathogenesis of Ancylostomatidae in humans and animals is a result of blood loss during the parasite’s feeding in the small intestine [24]. There is scarce knowledge on the pathogenesis of *G. urosubulatus*, specifically, previously published in the literature, but it is known that heavy infections can result in anemia [25].

## 5. Conclusions

We evidenced *G. urosubulatus* in all free-ranging wild boars investigated in our study. In relation to the morphological and morphometric characteristics of these helminths, we observed the asymmetry of the copulatory bursa for the first time in the literature. 

## Figures and Tables

**Table 1:** Ecological descriptors of parasitism by *Globocephalus urosubulatus* (Alessandrini 1909) recovered in 10 free-ranging wild boars in the northwest region of the State of São Paulo, Brazil.

Helminth	Habitat	Infection Indicators				
		I.	MI.	Ma.	Oc. (%)	TH.
*G. urosubulatus*	**SI.**	17–1.931	275	275	100%	2.750

***G.****—Globocephalus.* **SI.**—Small Intestine. **I.** –Intensity. **MI.**—Mean intensity. **Ma.**—Mean abundance. **Oc.**—Occurrence. **TH.**—Total Helminths.

**Table 2:** Morphometric comparison of *Globocephalus urosubulatus* (Alessandrini, 1909) recovered from 10 free-ranging wild boars in the northwest region of the State of São Paulo, Brazil.

Character	Present Study		[11]		[10]		[14]	
	Male	Female	Male	Female	Male	Female	Male	Female
**Length**	4.70–5.94	4.73–7.90	4.0–5.0	6.0–8.0	6.17 (471)	8.4 (0.027)	3.5–5.0	4.5–8.0
**Width**	286–342	423–583	167–300	429–514	350 (23)	470 (54)	360–370	420–500
**Bcs**	118–190	142–209	125–150	167–227	150 (14)	210 (17)	140–200	140–200
**Bcw**	95–125	137–163	100–140	140–160	130 (23)	140 (20)	150–170	150–170
**Esps**	512–624	671–795	487–540	593–687	620 (26)	840 (110)	560–690	560–690
**Espw**	112–145	141–200	93–133	147–173	-	-	120–150	120–150
**Nvr**	312–426	433–538	317–367	387–500	480 (39)	630 (35)	380–520	380–520
**Ep**	407–531	409–645	317–417	433- 547	490 (40)	650 (20)	-	-
**De**	387–568	440–652	370–533	433–547	560 (42)	710 (17)	430–610	430–610
**Vu**	-	1.48–3.0	-	3.0–5.0 *		6.67 (0.023)	-	2.20–2.40
**Ss**	444–573	-	337–527	-	610 (90)	-	420–580	-
**Gu**	60–86	-	60–88	-	80 (1)	-	70–80	-
**Anu**	-	76–238	-	130–200	-	220 (34)	-	120–180
**Mu**	-	25–79	-	Absent	-	-	-	40

**Bcs**—Buccal capsule size (µm). **Bcw**—Buccal capsule width (µm). **Esps**—Esophagus size (µm). **Espw**—Esophagus width (µm). **Nvr**—Distance from the nervous ring to the beginning of the anterior region (µm). **Ep**—Distance from the excretory pore to the beginning of the anterior region (µm). **De**—Distance from deirids to the beginning of the anterior region (µm). **Vu**—Distance from the vulva to the end of the posterior region (mm). **Ss**—Spicule size (µm). **Gu**—Gubernaculum size (µm). **Anu**—Distance from the anus to the end of the posterior region (µm). **Mu**—Tail size [mucrun] (µm). *—Distance from the vulva to the beginning of the anterior region (mm).

## Data Availability

Data is contained within the article.
